# Role of Protein Phosphorylation and Tyrosine Phosphatases in the Adrenal Regulation of Steroid Synthesis and Mitochondrial Function

**DOI:** 10.3389/fendo.2016.00060

**Published:** 2016-06-09

**Authors:** Cristina Paz, Fabiana Cornejo Maciel, Alejandra Gorostizaga, Ana F. Castillo, M. Mercedes Mori Sequeiros García, Paula M. Maloberti, Ulises D. Orlando, Pablo G. Mele, Cecilia Poderoso, Ernesto J. Podesta

**Affiliations:** ^1^Departamento de Bioquímica Humana, Facultad de Medicina, Instituto de Investigaciones Biomédicas (INBIOMED), Universidad de Buenos Aires (UBA-CONICET), Ciudad Autónoma de Buenos Aires, Argentina

**Keywords:** PKA, PTPs, ERK1/2, SHP2, mitochondrial dynamics, MKP-1, Acsl4

## Abstract

In adrenocortical cells, adrenocorticotropin (ACTH) promotes the activation of several protein kinases. The action of these kinases is linked to steroid production, mainly through steroidogenic acute regulatory protein (StAR), whose expression and activity are dependent on protein phosphorylation events at genomic and non-genomic levels. Hormone-dependent mitochondrial dynamics and cell proliferation are functions also associated with protein kinases. On the other hand, protein tyrosine dephosphorylation is an additional component of the ACTH signaling pathway, which involves the “classical” protein tyrosine phosphatases (PTPs), such as Src homology domain (SH) 2-containing PTP (SHP2c), and members of the MAP kinase phosphatase (MKP) family, such as MKP-1. PTPs are rapidly activated by posttranslational mechanisms and participate in hormone-stimulated steroid production. In this process, the SHP2 tyrosine phosphatase plays a crucial role in a mechanism that includes an acyl-CoA synthetase-4 (Acsl4), arachidonic acid (AA) release and StAR induction. In contrast, MKPs in steroidogenic cells have a role in the turn-off of the hormonal signal in ERK-dependent processes such as steroid synthesis and, perhaps, cell proliferation. This review analyzes the participation of these tyrosine phosphates in the ACTH signaling pathway and the action of kinases and phosphatases in the regulation of mitochondrial dynamics and steroid production. In addition, the participation of kinases and phosphatases in the signal cascade triggered by different stimuli in other steroidogenic tissues is also compared to adrenocortical cell/ACTH and discussed.

## Introduction

Steroid hormones are synthesized in steroidogenic cells of the adrenal gland, ovary, testis, placenta, and brain and are required for normal reproductive function and body homeostasis. Unlike cells producing polypeptide hormones, which store large amounts of hormone in secretory vesicles ready for rapid release, steroidogenic cells store low amounts of steroids. Thus, a rapid steroidogenic response requires a rapid synthesis of new steroid molecules.

The transport of cholesterol from the outer to the inner mitochondrial membrane (IMM) is the rate-limiting step of steroidogenesis (1, 2), and it is controlled by a complex mechanism that includes phosphorylation–dephosphorylation processes and the interaction of several proteins. Among these, the steroidogenic acute regulatory protein (StAR along this review, also known as STAR or more precisely STARD1) is the most widely studied (3, 4). Indeed, specialized reviews have focused and deeply covered the role of StAR protein in steroidogenesis (3, 4).

Steroid biosynthesis is finely regulated by the phosphorylation–dephosphorylation of intermediate proteins (5–9). In this regard, it is well accepted that steroidogenic hormones act through the activation of serine/threonine (Ser/Thr) protein kinases. In all steroidogenic tissues, phosphorylation-dependent events are required for the acute stimulation of steroid biosynthesis through the activation of protein kinases, including cAMP-dependent protein kinase (PKA), protein kinase C (PKC), calcium/calmodulin-dependent protein kinase, and mitogen-activated protein kinases (MAPKs). Adrenocorticotropic hormone (ACTH) and luteinizing hormone (LH) [or its surrogate chorionic gonadotropin (CG)] signal transduction pathways include PKA-dependent phosphorylation events in adrenal and Leydig cells, respectively (10–12) In the adrenal zona glomerulosa, aldosterone secretion is stimulated by angiotensin II (Ang II) and K^+^, in addition to ACTH. These stimuli promote phosphorylation events, which are not dependent on cAMP/PKA. Indeed, K^+^ activates voltage-operated Ca^2+^ channels, while Ang II, bound to Ang II type 1 receptors, acts through the inositol 1,4,5-trisphosphate IP_3_–Ca^2+^/calmodulin system (13). In other words, steroid biosynthesis is modulated by hormones, ions, or growth factors through the posttranslational phosphorylation of proteins, while the question that arises is how these phosphorylation events can lead a specific signal to its mitochondrial site of action.

Signal transduction pathways in eukaryotic cells include protein phosphorylation as an integral component regulated by the delicate balance between protein kinases and phosphatases activity. Thereby, many cellular responses require a coordinated cross talk between Ser/Thr and Tyr kinases and phosphatases activity.

In this context, this article will discuss the role of protein phosphorylation–dephosphorylation in cellular biology and endocrine function of steroidogenic cells.

## Protein Phosphorylation

### Kinases Involved in StAR Phosphorylation

Since the middle 80s, Orme-Johnson and her group were pioneers describing the relevance of the rapid induction of a 30-kDa protein in adrenal cortex after ACTH stimulation (14) and Leydig cells stimulated by cAMP (15). After these first discoveries, this group demonstrated that this protein is accumulated in mitochondria after hormone stimulation and processed to render two isoforms of 32 and 30 kDa (16, 17). Later, Stocco and Clark provided important data on the crucial role of this protein on the acute regulation of steroidogenesis and also on its molecular aspects. Since this protein is essential for cholesterol transport to the IMM and consequently for steroid synthesis, it was named StAR (3). Even when StAR has been widely identified as a phosphoprotein, the exact role of phosphorylation in StAR protein activity and hence cholesterol transport to the IMM still remains to be fully elucidated.

It is well established that non-genomic effects of PKA mainly involve posttranslational modifications of StAR protein. In fact, PKA phosphorylates murine and human StAR at specific residues such as Ser56/57 and Ser194/195 (18, 19). Moreover, genomic effects of PKA are known to include not only *STAR* gene (also known as *STARD1* gene) transcription but also the transcriptional regulation of several steroidogenic-related genes (20). Even if cAMP-dependent signaling is the major pathway in steroid biosynthesis stimulated by ACTH and LH/CG, and PKA phosphorylation sites in StAR protein are well described, it is noteworthy that StAR sequence also contains putative phosphorylation sites for PKC, cGMP-dependent protein kinase or protein kinase G (PKG), casein kinase I and II, and cyclin-dependent kinase 5 (Cdk5), as it was described elsewhere for eukaryotic phosphoproteins using database Expasy Prosite^1^ (21). Although the presence of these consensus sites might indicate StAR as a possible substrate for the respective kinases, the occurrence of this phosphorylation *in vivo* and its impact on steroid production remain uncertain.

Recent studies by Sasaki et al. – using a transgenic model with a bacterial artificial chromosome expressing either wild-type (WT) StAR or mutant StAR S194A to rescue StAR knockout mice – have demonstrated that Ser194, a conserved site among species, is an essential residue for normal StAR function in mice adrenal cortex and testis (22). These data indicate that phosphorylation of the Ser194/195 residues of StAR may account, at least in part, for the immediate increase in cholesterol side chain cleavage as a result of enhanced StAR protein activity. Consistent with these results, it has been demonstrated that the mutation in Ser195 in human mature StAR protein, which lacks the leader peptide, reduces pregnenolone production, as determined by an *in vitro* assay using mitochondria isolated from MA-10 Leydig cells (23). Strikingly, when cholesterol binding to StAR is measured with fluorescent or radioactive cholesterol, purified mutant S195A and WT StAR display equal binding activity. As determined by StAR structural analyses, Ser195 lies in a short loop opposite to the C-α helix, which is essential for cholesterol binding. Therefore, the addition of phosphate-negative charge in this Ser might influence StAR activity by modifying its interaction with hypothetical mitochondrial partners such as ACBD3 (previously known as peripheral benzodiazepine receptor-associated protein, PAP7) (24). This could, in turn, anchor PKA to Ser194/195, rather than alter cholesterol binding to the sterol-binding pocket (23).

In the same line, work by Stocco’s group has explored the regulation mechanism of StAR expression and steroidogenesis in conjunction with PKA and PKC pathways in MA-10 Leydig cells (25). This study shows that PKC-dependent induction of steroid synthesis is low when compared to that observed with PKA signaling, but it is capable of enhance LH/CG- and/or cAMP-stimulated steroidogenic response. On the one hand, the activation of PKC markedly increases StAR expression, but not phospho-StAR, with only a modest increase in progesterone production. On the other hand, PKA activation triggers a substantial increase in the band of StAR phosphorylated in Ser194 (25).

### Role of ERKs in the Regulation of StAR Protein and Steroid Biosynthesis

In addition to PKA activation importance for trophic hormone-stimulated steroid biosynthesis, it is also known that extracellular signal-regulated kinases 1 and 2 (ERK1/2) and upstream activator mitogen-activated protein kinase kinase 1 and 2 (MEK1/2) participate in the regulation of steroidogenesis. Indeed, several reports describe the role of members of the MAPK family in the regulation of steroid synthesis acting at both genomic and non-genomic levels.

One of the first published works in the field indicates that cAMP-induced steroid synthesis depends on ERKs phosphorylation and activation (26). These authors show that adenylyl cyclase activation with forskolin promotes a time-dependent increase in ERK activity and translocation of this enzyme to the nucleus in mouse adrenocortical Y1 cells. Similarly, Roy et al. have demonstrated that ACTH receptor activation leads to rapid ERK1/2 phosphorylation in primary cultures of human fasciculata cells (27), an effect also observed in a human adrenocortical H295R cell line (28). Moreover, Ang II also promotes MAPK activation in adrenal glomerulosa cells (29, 30). Thus, ERK activation seems to be a common event in the stimulation of different steroidogenic systems.

Although already demonstrated, PKA involvement in ERK activation continues to generate controversy; Le and Shimmer have shown that ACTH increases MEK and ERK phosphorylation in Y1 adrenocortical murine cells. This effect has also been detected in Kin-8 cells, a PKA-deficient mutant Y1-derived cell line (31), which suggests that ERK activation is independent of PKA activity. H295R adrenocortical cells exhibit only a very modest cAMP response to ACTH, yet ERK1/2 response is immediate and consistent. ERK activation is minimally reduced by PKA inhibitor H89, but unaffected by PKC and calcium inhibitors. Thus, ACTH-induced ERK1/2 activation in H295R cells does not appear to depend on the mechanism by which most G protein-coupled receptors activate ERK1/2, but does seem to depend on receptor internalization (28). On the other hand, Roy et al. have demonstrated the participation of PKA in ERK activation in human fasciculata cells (27).

A role for ERK activity has also been demonstrated in adrenal and gonadal steroidogenesis. Gyles et al. have shown that ERK activation results in enhanced phosphorylation of steroidogenic factor 1 (SF-1) and increased steroid production through increased transcription of the *STAR* gene in Y1 cells (26). The activation of the ERK/MEK pathway correlates with an increase in StAR mRNA levels, StAR protein accumulation, and steroidogenesis. Similarly, ERK inhibition leads to a reduction in the levels of forskolin-stimulated StAR mRNA, StAR protein, and steroid secretion (26).

Luteinizing hormone receptor cascade activation in Leydig cells also promotes ERK1/2 phosphorylation, which is mediated by PKA through Ras activation (32). More recently, and using mice with a Leydig-specific deletion of MEK1/2 as an experimental model, Yamashita et al. have concluded that the MEK/ERK pathway is critical for maintaining a functional population of adult Leydig cells and fertility (33).

In agreement with findings in adrenocortical cells (26), Martinelle et al. have demonstrated the functional role of the ERK cascade in human CG (hCG)-induced steroidogenesis in primary cultures of immature rat Leydig cells (34). In this system, inhibition of MEK1/2 by U0126 suppresses several cellular responses to hCG.

In turn, 3-day treatment with Ang II in cultured rat adrenal glomerulosa cells increases aldosterone secretion through a mechanism involving both ERK1/2 and p38 MAPK pathways (30). In addition, the effect of Ang II on aldosterone synthesis also requires ERK1/2 activity in primary cultures of glomerulosa bovine cells (29).

Even though several reports support a role for ERK1/2 in StAR mRNA induction and steroid biosynthesis, other studies show controversial results. Indeed, it has been demonstrated that MEK1/2 inhibitors, such as U0126 and PD98059, enhance the expression of StAR protein in MTLC-1 and primary Leydig cells (35). Also, in MA-10 Leydig cells stimulated with dibutyryl cAMP, inhibition of ERK1/2 activity increases *STAR* gene expression (25). Similarly, Seger et al. have demonstrated that ERK signaling cascade inhibits CG-stimulated steroidogenesis in granulosa-derived cell lines (36). Taken together, the discrepancies on the role of ERK1/2 in StAR transcription might be due to different experimental conditions and cellular types, which generate different factor availability, such as transcription factors required for StAR expression. Nevertheless, the results of Yamashita et al. strongly support the requirement of ERK for StAR expression and steroidogenesis (33). Indeed, they analyzed the role of ERK1/2 on steroidogenesis and fertility using as experimental models knockout mice carrying a deletion for MEK1/2 in Leydig cells and primary culture of Leydig cells isolated from these knockout mice. This study demonstrates that the deletion of MEK1/2 and concomitant reduction of phospho-ERK1/2 levels decreased testicular expression of several Leydig cells markers, including StAR protein. Then, a similar experimental model based on transgenic mouse should be a powerful tool to univocally demonstrate the role of ERK1/2 on ACTH action on steroidogenesis and cell growth.

Extracellular signal-regulated kinase activity seems to regulate key steroidogenic transcription factors by non-genomic and genomic actions. *STAR* gene transcriptional regulation requires transcription factors already present in the cell, which are activated by posttranslational modifications, such as SF1, and others which must be *de novo* synthesized, e.g., NUR77, encoded by *Nr4a1* gene (37). Finally, it has been pharmacologically and molecularly demonstrated that ERK1/2 participates in cAMP-induced *Nr4a1* expression in both MA-10 Leydig and Y1 adrenocortical cells (38), in addition to SF-1 activation (26).

In addition to their role in steroid biosynthesis, ERK1/2 is also involved in adrenal cell proliferation and growth (39–41). ACTH stimulates adrenal growth *in vivo*, whereas *in vitro* ACTH has an inhibitory effect on adrenal cell proliferation. In serum-starved Y1 cells, a short pulse of ACTH produces a mitogenic effect, which is preceded by the rapid activation of ERK1/2 (39). This result is in accordance with the requirement of ERK activation for cell proliferation. However, it is well documented that ACTH-mediated ERK activation is a transient process in Y1 cells. Thus, the early ERK activation could trigger StAR induction, steroidogenesis, and also cell proliferation, while the following decrease in pERK levels could contribute to the inhibition of cell proliferation mediated by prolonged exposure to ACTH.

### ERK-Mediated Phosphorylation of StAR Protein

The activation of the MEK1/2–ERK1/2 cascade appears to enhance steroid synthesis; nevertheless, the requirement of MEK1/2 and ERK1/2 cascade for the induction of *STAR* gene expression is less evident. Although it is well known the regulatory role of PKA on StAR protein activity, also this MEK1/2 and ERK1/2 cascade has been unveiled as a new mechanism of StAR activity modulation.

Our group has reported the role of MEK1/2–ERK1/2 cascade in the hCG/LH stimulation of StAR protein activity and steroidogenesis (42). In line with reports by Manna et al. and Martinelle et al. (25, 34), our work has shown that PKA acts upstream the stimulation of MEK and ERK activities. The inhibition of MEK1/2 on stimulated progesterone synthesis is not mediated by inhibition of PKA, as this enzyme activity is not altered in the presence of both inhibitors, U0126 and PD98095 (42).

Using a different strategy to study the role of active ERK1/2 in steroidogenesis, the overexpression of a WT form of ERK2 in MA-10 Leydig cells was performed. We observed an increase in steroid production stimulated by submaximal concentration of cAMP (42). Furthermore, an inactive form of ERK2, the H230R variant, which fails to interact with MEK1, but retains the ability to interact with MEK2 in a weakened fashion, does not produce the effect of WT ERK2 (42).

In short, both kinases, PKC and PKA, are capable of phosphorylating ERK1/2 through MEK1/2 activation (25, 32). ERK1/2 activity is involved in *STAR* gene expression induced by PKC or PKA activation, while a relevant role in StAR protein phosphorylation is attributed to PKA signaling pathway (25).

In summary, it is recognized that the ERK1/2 signaling cascade involved in regulating StAR expression and steroid synthesis is mediated by multiple factors and pathways, and is stimulus-specific.

### MEK1/2 and ERK1/2 at the Mitochondria

The site of action of MEK inhibitors appears to be downstream of PKA activation and before of cholesterol transport, which implies that one of the targets may be located at the mitochondria. Gyles et al. have observed that activation of adenylyl cyclase causes a time-dependent increase in ERK activity and its localization from cytoplasm to nucleus (26), and our group has further proven a temporal ERK1/2 activation localized in the mitochondria, which is obligatory for PKA-mediated steroid synthesis in Leydig cells (42). Worth pointing out, the phosphorylation of mitochondrial ERK occurs before the increase in steroid production, and the hormone dose that is required for ERK activation at the mitochondria is the equivalent for eliciting steroid synthesis. Phosphorylated ERK1/2 (pERK1/2) is located in the cytosol, mitochondria, and, in lower proportion, in the nuclear fractions after cAMP stimulation. In the mitochondria and the cytosol, an early peak in ERK1/2 phosphorylation is followed by a slow progressive signal reduction during the first hour of cAMP incubation, a profile similar to that observed in hCG stimulation, leading to pERK1/2 activation. In contrast, pERK1/2 is mainly localized in the cytosol and nucleus, after epidermal growth factor (EGF) stimulation. Two different pools of MEK1/2 and pMEK1/2 have been found to be constitutively present in the cytosol and mitochondria. Remarkably, MEK1/2 differential distribution triggers different responses upon cellular stimulation (42).

Poderoso and coworkers have also shown that cAMP clearly induces sustained MEK1/2 phosphorylation in mitochondria, with a minor effect on the cytosolic kinases. Conversely, EGF induces a prolonged and strong cytosolic MEK1/2 activation, but only a discrete phosphorylation, in mitochondria. Although both EGF and cAMP increase total cytosolic MEK1/2, only EGF promotes its phosphorylation in this subcellular fraction (42).

The inhibition of PKA activity with the compound H89 and by PKA knockdown experiments diminishes the increase in mitochondrial pMEK1/2 and pERK1/2 after cAMP action (42). In agreement, the increase in mitochondrial PKA activity occurs after 5 min of cAMP action in parallel with the appearance of the phosphorylated forms of MEK1/2 and ERK1/2 in this organelle.

In regard to PKA activity and subcellular organization, a family of proteins named A-kinase anchor proteins (AKAPs) enhances cAMP-dependent pathways (43, 44). AKAPs raise cAMP signal by anchoring PKA near its cellular substrate, while mouse-derived AKAP121 binds PKA to the mitochondrial outer surface (45, 46). In addition, purified AKAP121 KH domain binds the 3′-untranslated regions of transcripts encoding the Fo-f subunit of mitochondrial ATP synthase and manganese superoxide dismutase (47). A special member of the AKAP family, AKAP121, can be anchored to mitochondria and may compartmentalize PKA and other proteins on the outer mitochondria membrane (OMM) (48). In Leydig cells, cAMP-induced StAR expression and steroidogenesis were found to correlate with the extent of AKAP 121 expression (49). Expression and role of AKAP121 in H295R cells deserve elucidation. Another relevant AKAP in steroidogenic tissues is the ACBD3 protein, an acyl CoA-binding protein, known previously as PAP7 (24). Human ACBD3 is highly expressed in steroidogenic tissues, where it follows the pattern of PRKAR1A expression, suggesting that it participates in PRKAR1A-mediated tumorigenesis and hypercortisolism (50). Therefore, StAR protein is likely to be phosphorylated at the mitochondria by the activation of a cascade of kinases, including ERK.

### StAR Protein as a Substrate of ERK1/2

Steroidogenic acute regulatory protein structural analyses have revealed a consensus sequence that would allow the docking of StAR protein to ERK1/2 and a consensus site for ERK1/2 phosphorylation. A typical docking site known as the D domain (**K**T**K**LTWL**LSI**) lies between amino acids 235 and 244 and is conserved among MEK1/2, MAPK phosphatase, and ERK substrates (51). Regarding the ERK1/2 phosphorylation site in StAR protein, it was possible to detect only two Ser-Pro motifs, at Ser232 and Ser277, targets for ERK1/2 phosphorylation in the mature form of the murine StAR protein. In accordance with the database Expasy Prosite,^2^ Ser232 (PLAGS^232^PS) has a 90% probability of phopshorylation and is adjacent to the docking D domain, while the probability of Ser277 is only 5%. Besides, Ser277 is relatively less conserved among species. In agreement of a predicted StAR–ERK binding, the treatment of subcellular fractions with pERK–GST has shown that StAR protein interacts with pERK1 just in the mitochondrial fraction, but not in the cytosol. Together, MEK phosphorylation PKA-dependent, mitochondrial StAR and pERK1/2 activity increase cholesterol transport and mitochondrial synthesis of progesterone in cell-free assays (42).

*In vitro* phosphorylation assays using recombinant 30-kDa form of StAR protein and WT and the inactive mutant K71A forms of ERK1 demonstrated that the StAR protein is indeed phosphorylated by ERK1 and not by the K71A mutant. Remarkably, phosphorylation of StAR by ERK1 is dependent on the presence of cholesterol, while phosphorylation by PKA is not. Besides, StAR phosphorylation by PKA does not require previous ERK phosphorylation. By means of directed mutagenesis of Ser 232 (S232A), we demonstrated that this residue is indeed the target of ERK (42).

Expression of S232A mutated form of StAR partially blocks progesterone production enhanced by cAMP treatment in MA-10 cells. In contrast, the StAR mutant in which Ser 232 is replaced by a glutamic acid (S232E) does not produce such effect, which suggests that the negatively charged amino acid partially mimics the negative charge of the phosphate group present in the phospho-Ser (42).

Taking together, PKA phosphorylates StAR protein and also activates mitochondrial MEK1/2. Then, phosphorylated MEK1/2 activates a non-phosphorylated mitochondrial pool of ERK1/2 when the three kinases interact at the OMM, a crucial site for cholesterol transport forming a mitochondrial multi-complex with StAR.

## Protein Dephosphorylation

### Regulation of Protein Tyrosine Phosphatases by Steroidogenic Hormones

The degree of tyrosine phosphorylation of a given protein is the result of the action of protein tyrosine kinases and protein tyrosine phosphatases (PTPs). Protein kinases have been the focus of the research for a long time. Proportionately much less research has focused on protein phosphatases.

Whereas PTPs were initially regarded as household enzymes with constitutive activity and capable of all-substrate dephosphorylation, evidence in favor of tight regulation of PTP activity by various mechanisms is now accumulating. Like protein phosphorylation, dephosphorylation by PTPs is required in a cell compartment-specific manner. Protein–protein interaction domains and compartment-specific targeting domains in PTPs serve to localize PTPs all over the cell compartments (52).

Based on the amino acid sequence of their catalytic domain, PTPs are classified into four groups, Class I, II, III (all Cys-based PTPs), and IV (Asp-based PTPs), each with a specific range of substrates. The largest family is the Class I (Cys-based PTPs), comprising the 38 “classical” PTPs, and the 61 “dual-specificity” PTPs (DSPs), which is the most diverse group in terms of substrate specificity. The group of the classical PTPs, with strict tyrosine specificity, consists of the receptor-like (transmembrane) and non-receptor (intracellular) classes. In the human genome, these PTPs comprise 21 and 17 genes, respectively (52, 53).

The cross talk between pathways that involve Ser/Thr phosphorylation and Tyr dephosphorylation has been described in the regulation of steroid synthesis. Our group has reported that ACTH treatment causes an increase in the activity of PTPs located in the cytosol of adrenal zona fasciculata (ZF). The stimulation is detected very soon after ACTH stimulation (5 min), reaches a maximum (twofold) after 15 min, and returns to basal levels after 30 min (54). Incubation of adrenal ZF with 8Br-cAMP (permeant analog of cAMP) also produces PTPs activation, suggesting that it can be mediated by PKA-dependent phosphorylation. Moreover, detection of PTP activity by in-gel assays has shown at least two ACTH-stimulated soluble PTPs with molecular masses of 115 and 80 kDa (54).

Protein tyrosine phosphatases are regulated by Ser/Thr or Tyr-kinases. Indeed, several PTPs are known to be phosphoproteins *in vivo* (55, 56), which reflects the potential of cross-regulation between kinases and phosphatases, either PTPs or Ser/Thr phosphatases, for the fine control of cellular activity. Among those phospho-PTPs, there is a membrane-bound form, which can be activated by treatment of intact cells with isoproterenol, forskolin, or cAMP analogs (55), and a soluble form of PTP, known as PTP-PEST, which is inhibited after *in vitro* phosphorylation by PKA and in HeLa cells after forskolin or methylisobutylxanthine treatment (56). Our current studies demonstrate the expression of PTP-PEST in Y1 cells and in rat adrenal ZF and suggest that the ACTH-activated PTP of 115 kDa could be PTP-PEST. Furthermore, when paxillin is precipitated from the cytosol of ACTH-treated rats, a PTP of 115 kDa is coprecipitated according to the analyses of precipitate by in-gel PTP assay (57).

The Src homology domain (SH) 2-containing PTP (SHP2) is classified among the non-receptor, classical PTPs. It is widely expressed and plays an essential role in many organisms from lower eukaryotes to mammals (58). In contrast to other PTPs that inactivate intracellular signaling pathways, SHP2 activates them (59). The ACTH-activated PTP of 80 kDa from adrenal ZF has been recognized by a commercially available antibody against SHP2 in Western blot analyses (unpublished results), which suggests that SHP2 is a PTP activated by ACTH in rat adrenal ZF. In line with our results, Rocchi et al. have demonstrated SHP2 expression in bovine adrenocortical cells and its activation by ACTH through PKA-dependent phosphorylation (60). Finally, our group has also shown that SHP2 is expressed in MA-10 Leydig cells (61).

### PTP Activity and Steroidogenesis

The rapid increase in PTP activity induced by ACTH may prove that this activity is necessary in the stimulation of steroidogenesis. Studies on PTPs role in the acute steroidogenic response to hormones have been performed using incubation of rat adrenal ZF cells with two powerful cell permeant PTP inhibitors, phenylarsine oxide (PAO) and pervanadate (PV), and evaluation of the steroid production upon stimulation by ACTH and 8Br-cAMP. It has been proven that those PTP inhibitors block ACTH- and cAMP-stimulated corticosterone production, but exert no effects on basal steroidogenesis (54). Similar conclusions were obtained using Y1 cells (62, 63).

Phenylarsine oxide and PV also reduce LH/hCG- and cAMP-stimulated steroid production in testicular interstitial cells (64) and in MA-10 cells (65). PTP inhibitors affect StAR induction at the protein and mRNA levels in MA-10 Leydig cells (65), as well as in Y1 cells (63). PTP inhibitors affect neither cell viability nor mitochondrial enzymatic activity evaluated as steroidogenesis triggered by 22-OH-cholesterol treatment. Thus, hormone-dependent steroid synthesis requires PTP activity in a site localized beyond PKA actions and before cholesterol transport across the IMM.

Phenylarsine oxide oxidizes the thiol group of a cysteine present in the active site of all PTPs. Benzyl phosphonic acid (BPA) has a structure very similar to the PTP substrate, exerting its inhibitory action on PTPs by competitive inhibition of the enzyme. However, both inhibitors, PAO and BPA, inhibit Ang II- or K^+^-induced steroid synthesis in a dose-dependent fashion in H295R cells, a cell line derived from human ZG tumor (66), and in Y1 cells (63).

Collectively, our group’s work demonstrates that steroidogenic stimulus (ACTH, LH, Ang II, and K^+^), acting by different signal transduction pathways, conveys on PTPs as common intermediaries (63–66).

In regard of substrates downstream PTP activity, *in vivo* ACTH treatment decreases phosphotyrosine contents in several proteins, one of them identified as paxillin, a focal adhesion protein (54). In Y1 cells, ACTH and cAMP elicit a rapid morphological transition from a flat epithelioid morphology to rounded cells (11). cAMP causes a rapid and selective Tyr dephosphorylation of paxillin in these cells (67). Moreover, the inhibition of PTP activity blocks changes in cell shape promoted by ACTH (67). Taken together, these results indicate that PTP activity is involved in cAMP-dependent paxillin dephosphorylation and this might mediate hormone-stimulated cell shape changes in adrenocortical cells.

In summary, results presented here support the view that the morphological and functional responses to ACTH in adrenocortical cells are intimately linked to and mediated by PTP activity.

### Links between PTP Activity and Arachidonic Acid Release

cAMP- and PKA-dependent pathways triggered by trophic hormones in steroidogenic cells stimulate arachidonic acid (AA) release (68, 69). AA and its metabolites take part in the acute stimulation of steroid production. The effect is exerted on both the expression and function of StAR (70, 71). Previously, we proposed that free AA levels in steroidogenic cells are determined by a novel hormone-regulated mechanism (69, 72, 73). This mechanism involves the concerted action of an acyl-CoA synthetase (Acsl4) and an acyl-CoA thioesterase (Acot2). Acsl4 is a long chain fatty acid acyl-CoA synthetase, with high affinity for AA, and it is preferentially expressed in steroidogenic tissues (72, 74). Acot2, a thioesterase that acts on long chain fatty acyl-CoA, associates with the matrix face of mitochondrial cristae (75–77). Acot2 mRNA and protein are present in adrenal cortex, ovary, testis, placenta, and brain, among other tissues. The activity of both enzymes is acutely modified after hormone stimulation of steroidogenic cells. Acot2 is activated by phosphorylation and substrate availability (78), and Acsl4 is rapidly induced after hormone treatment (79).

The activity of Acsl4 and Acot2 are needed for AA release, StAR induction, and steroidogenesis. This statement is supported by the fact that the reduction of the expression of both, Acsl4 and Acot2, causes an inhibition of steroid production in two steroidogenic systems (79, 80). Moreover, this effect is overcome by addition of exogenous AA (80). On the basis of these results, we propose that upon hormone treatment, Acsl4 would convert free AA in AA–CoA. The action of mitochondrial Acot2 on AA–CoA would release AA, specifically in the mitochondria, to increase StAR and steroidogenesis (69).

**Figure 1:**
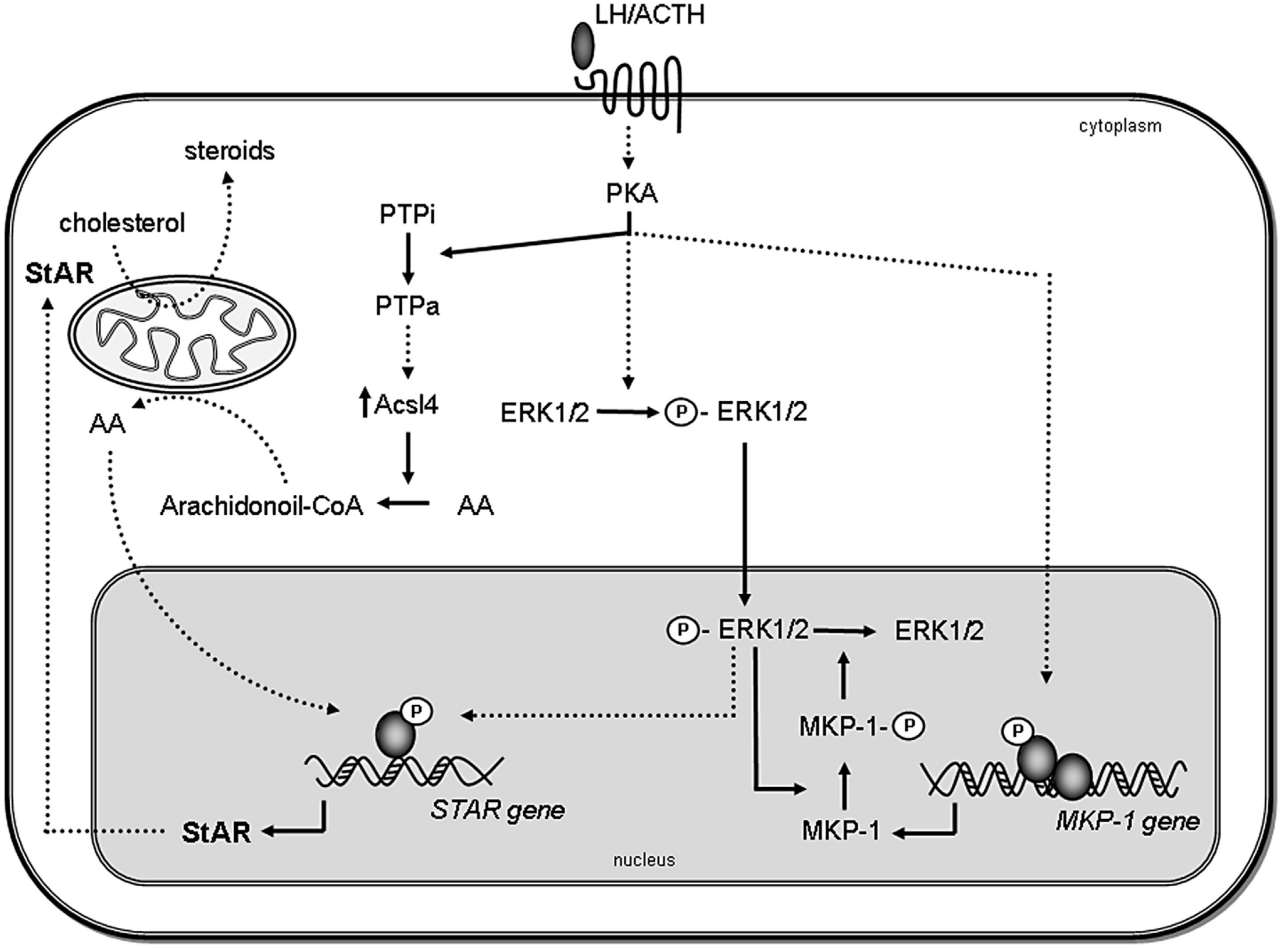
**Proposed model for the role of MAPK and phosphatases in steroidogenesis**. Steroidogenic hormones trigger the activation of PKA, which leads to the rapid phosphorylation of ERK1/2. Activated ERK (P-ERK) translocates to the nucleus, where it phosphorylates and activates transcription factors, leading to STAR gene induction. Then, StAR protein acts in the mitochondria facilitating steroid synthesis. Simultaneously, activated PKA also induces MKP-1 gene transcription. MKP-1 is stabilized by ERK-mediated phosphorylation; therefore, the stabilization promotes its accumulation in the cell. In turn, MKP-1 dephosphorylates ERK1/2, generating a negative feedback mechanism on its activity, thus terminating MAPK-regulated events involved in steroidogenesis. Inactive PTPs (PTPi) are activated by PKA (PTPa) and participate in Acsl4 regulation, which in turn, through arachidonic acid (AA) metabolism, leads to the increase of StAR gene expression. Direct effects are indicated by solid lines, whereas indirect effects are indicated as dotted lines.

Our group has also linked the sequential action of PTPs, Acsl4, and StAR to the hormone-stimulated steroid production (66, 81). In Y1 cells, inhibition of PTP activity prevents Acsl4 and StAR induction exerted by 8Br-cAMP (81). Moreover, the effect of PTP inhibition is overcome by addition of exogenous AA (81). These results indicate that there is a consecutive action of PTP and Acsl4 to release AA before StAR induction. Moreover, the effect of PTPs on Acsl4 is also described in Leydig (81) and adrenocortical ZG cells (66) (Figure 1), indicating that the action of PTPs on Acsl4 may be a regulatory event that controls the steroidogenesis.

These results brought about a challenge to determine the identity of the PTP involved in the stimulation of steroid synthesis through AA release.

### SHP2 Involvement in Steroid Synthesis

By means of overexpression and suppression approaches, SHP2 has been proven to be at least one of the PTPs playing an obligatory role in steroidogenesis. NSC87877, a specific inhibitor of the tyrosine phosphatase SHP2, has been shown to reduce Acsl4 protein levels in Acsl4-rich breast cancer cells and steroidogenic cells. In addition, overexpression of an active form of SHP2 has increased Acsl4 protein levels in MA-10 Leydig cells. SHP2 has to be activated through a cAMP-dependent pathway to exert its effect on Acsl4, which could be specifically attributed to SHP2, as phosphatase knockdown reduces Acsl4 mRNA and protein levels. Through the action on Acsl4 protein levels, SHP2 affects AA–CoA production and metabolism and, finally, the steroidogenic capacity of MA-10 cells: overexpression (or knockdown) of SHP2 leads to increased (or decreased) steroid production (82).

The downregulation of SHP2 also modifies StAR expression. StAR expression increases in MA-10 Leydig cells treated with cAMP, an effect impaired by a short hairpin RNA (shRNA) against SHP2. Also, cAMP treatment causes a significant increase in StAR levels in mock-transfected cells, whereas SHP2 shRNA treatment prevents this effect. The involvement of AA in this process receives strong support from the fact that AA addition to SHP2 shRNA-treated cells bypasses the inhibitory effect produced by SHP2 knockdown (61). In this context, the hypothesis that arises is that a putative transcription factor, inhibited by tyrosine phosphorylation, is involved in ACTH-mediated Acsl4 induction. SHP2 could promote the tyrosine dephosphorylation of this factor, thus increasing steroid synthesis.

The Src homology domain (SH) 2-containing PTP is phosphorylated upon ACTH treatment (60). Moreover, *in vitro* phosphorylation of SHP2 by PKA dramatically increases its phosphatase activity (60). Although the phosphorylation of SHP2 by cAMP-independent kinases has not been demonstrated in steroidogenic cells, SHP2 may be phosphorylated by several different kinases to become Ser/Thr- or Tyr-phosphorylated. Indeed, SHP2 itself is Tyr-phosphorylated and activated through the action of different growth factors (83–86).

The Src homology domain (SH) 2-containing PTP has been identified in mitochondria, being the first evidence of the presence of a tyrosine phosphatase in such organelle (87). Several tyrosine kinases are present in the mitochondria (88). Particularly, c-Src is involved in the modulation of the efficiency of the mitochondrial electron transport chain (88). These data become important as stimulation of steroidogenesis needs energized mitochondria, and AA export and StAR induction need the activity of complexes III and V (73). In case, SHP2 is also located in the mitochondria of steroidogenic cells, this phosphatase would be involved in the regulation of mitochondrial respiration.

**Figure 2:**
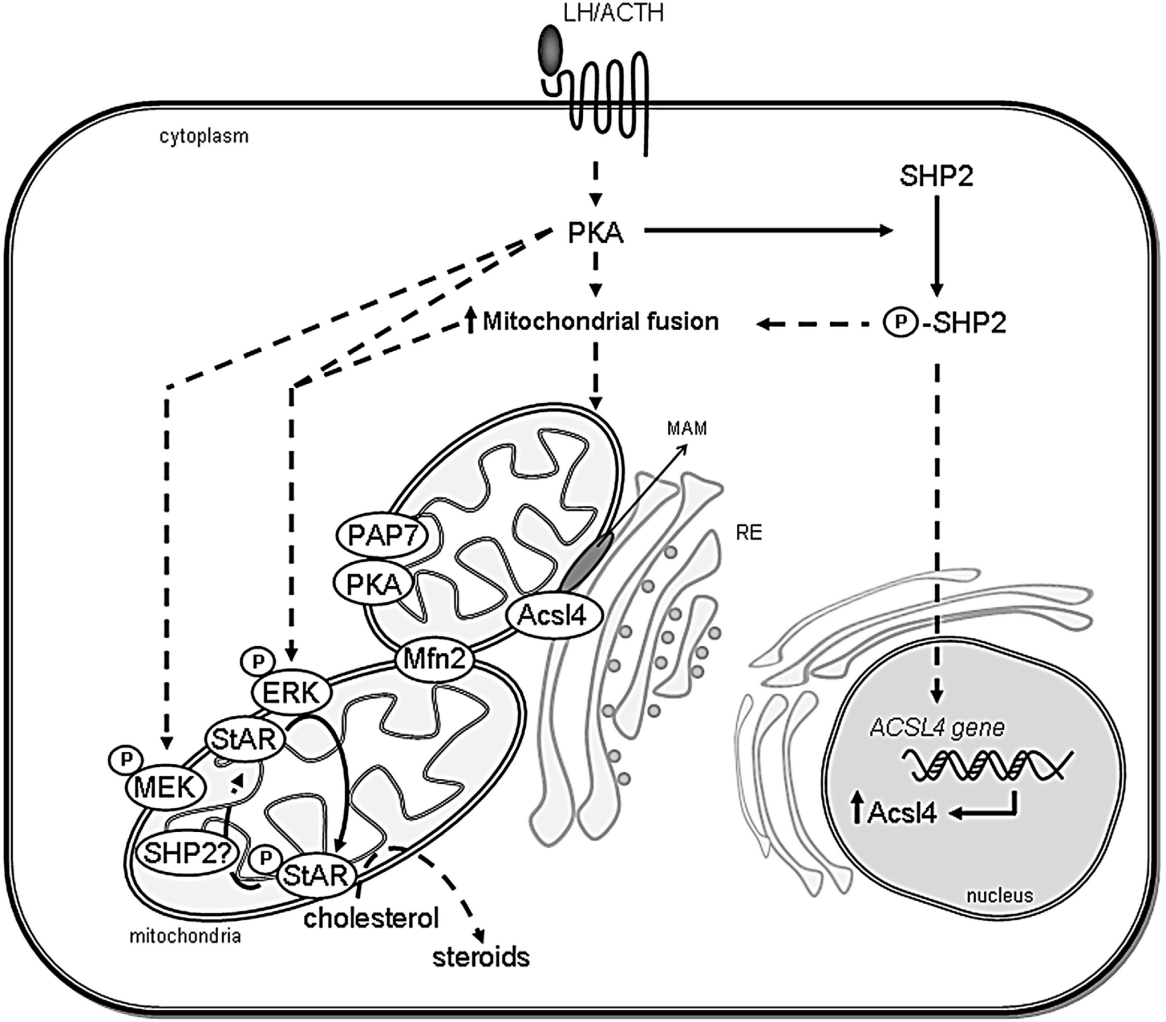
**Scheme showing the association between ER and mitochondria in steroidogenic cells**. The proposed interaction between organelles and the involved proteins in the regulation of cholesterol delivery is shown. After steroidogenic hormone action, PKA is activated and Mitofusin 2 (Mfn2) is localized in mitochondria. SHP2 is phosphorylated (P-SHP2) by active PKA, then it participates in both ACSL4 gene induction and the increase of mitochondrial fusion. Next, Acsl4 is localized in MAM subdomains in an Mfn2-dependent manner to exert its enzymatic activity. Mitochondrial ERK is activated by phosphorylation (P-ERK) in a PKA- and MEK-dependent mechanism and phosphorylates mitochondrial StAR (P-StAR) to achieve maximal steroid production. A putative mechanism involves StAR dephosphorylation by SHP2 in mitochondria to release a cholesterol molecule to be substrate of P450scc. Direct effects are indicated by solid lines, whereas indirect effects are indicated as dotted lines.

The translocation of ERK to mitochondria is abolished by SHP2 knockdown in MA-10 cells. Moreover, the pronounced rearrangement of mitochondria that occurs after hCG stimulation is reduced by the downregulation of SHP2 expression (89). Then, the complete description of the steroid synthesis and secretion after hormone stimulation needs the study of SHP2 activation and mitochondrial reorganization (Figure 2).

### MAPK Phosphatases in Steroidogenic Cells

Given that MAPK activation depends on Thr/Tyr protein phosphorylation, the magnitude and duration of their activity are related to protein phosphatases. MAPK phosphatases (MKPs) are a family of dual activity (Thr/Tyr) protein phosphatases, which dephosphorylate specifically members of MAPKs (90, 91). Several distinct mammalian MKP family members have been identified and characterized and can be divided into two broad classes. One group, typified as MKP-1, comprises nuclear enzymes rapidly induced by growth factors or stress signals. This group also includes MKP-2, a nuclear enzyme induced by the same stimuli that induce MKP-1, but with a slower kinetics. The second group, typified as MKP-3, includes predominantly cytosolic enzymes, and their transcripts are induced with delayed kinetics by specific stimuli, but not by environmental stress.

On the basis of evidence showing that ACTH can regulate the activity of MAPKs (31, 92), the regulation of MKPs by this hormone is expected. Analyses on MKP-1 induction in serum-starved Y1 cells demonstrated that ACTH stimulation results in a transient increase in MKP-1 mRNA followed by an increase in protein levels (93). Sewer and Wateman have also described the regulation of MKP-1 expression by cAMP in H295R cells. In this regard, they demonstrated that MKP-1 mRNA and protein levels are induced by cAMP, and overexpression of this phosphatase stimulates hCYP17 reporter gene activity. Besides, this study also demonstrates that PKA phosphorylates MKP-1 (94).

The hormone-dependent expression of MKP-1 has also been analyzed in MA-10 Leydig cells, where hCG/cAMP rapidly increases MKP-1 gene induction in a transient manner (95). Besides, MKP-1 protein levels increased in both nuclear and mitochondrial compartments. Moreover, MKP-1 increase (95) and ERK1/2 dephosphorylation in the mitochondria (42) are temporally coordinated events. In addition, our group has demonstrated that, in cells expressing flag-MKP-1 protein, hCG/cAMP trigger the phosphorylation and the accumulation of the recombinant protein in a time-dependent manner. Altogether, these results indicate that hCG modulates MKP-1 expression by transcriptional and posttranslational actions.

The functional role of MKP-1 in the regulation of steroidogenesis has also been analyzed in MA-10 Leydig cells. Work by our group demonstrates that MKP-1 overexpression downregulates the effects of cAMP on phospho-ERK1/2 levels, StAR expression, and steroidogenesis, while MKP-1 downregulation produces opposite effects. In summary, these data demonstrate that in Leydig cells, MKP-1 expression is regulated at multiple levels as a negative feedback regulatory mechanism to modulate the hormonal action on ERK1/2 activity and steroidogenesis (95).

Casal et al. have demonstrated the expression and regulation of MPK-1 also in primary cultures of bovine adrenal glomerulosa cells (29). These authors show that Ang II markedly increases MKP-1 protein levels in a time- and concentration-dependent manner. Ang II-induced phosphorylation of ERK1/2 leads to MKP-1 phosphorylation and, in turn, MKP-1 promotes ERK1/2 dephosphorylation. MKP-1 overexpression in bovine adrenal glomerulosa cells results in decreased phosphorylation of ERK1/2 and aldosterone production in response to Ang II stimulation. These results strongly suggest that MKP-1 is induced by Ang II and that it is involved in the negative feedback mechanism, ensuring adequate ERK1/2-mediated aldosterone production in response to the hormone.

In MA-10 Leydig cells, LH receptor stimulation also induces MKP-2 (96) and MKP-3 (97) through multiple mechanisms. While MKP-2 completes the ERK1/2 dephosphorylation in the nucleus initiated by MKP-1, MKP-3 dephosphorylates ERK1/2 in the cytoplasm.

In conclusion, stimuli promoting MAPK activity also regulate MKPs expression at multiple stages as a negative feedback regulatory mechanism to modulate hormonal actions on ERK1/2 activity and steroidogenesis (Figure 1).

## Kinases and Phosphatases in the Regulation of Mitochondrial Dynamics: Role in StAR Activity and Steroidogenesis

### StAR Structural Changes

Several protein kinases, such as PKA, MEK, and ERK – which are essential to complete steroidogenesis – form a mitochondria-associated complex and are completely required for mitochondrial cholesterol transport along with StAR and other proteins such as Acsl4, voltage-dependent anion channel (VDAC1), and adenine nucleotidetranslocase (ANT) (42, 98).

The precise mechanism of StAR action has been widely explored, but still remains elusive. StAR is synthesized as a 37-kDa protein with a typical mitochondrial leader sequence that directs the protein to the mitochondria for the import and cleavage to an intramitochondrial form of 30-kDa (99–102). After reaching the matrix, the 30-kDa StAR is controlled by the ATP-dependent Lon protease (103) and proteolysed, its half-life being 4–5 h (101, 104). A tight regulation of mitochondrial StAR levels is imperative since excessive accumulation of StAR protein in the matrix provokes mitochondrial damage and a “mitochondria to nucleus” signaling which, in turn, activates transcription of genes that encode mitochondrial proteases crucial for complete clearance of StAR (105). In this regard, we have observed that the presence of mitochondrial ERK is strictly necessary for protecting StAR from unknown proteases to avoid further degradation, which constitutes one of the mechanisms playing a role in mitochondrial StAR levels regulation (106).

This mechanistic model of StAR action suggests that the active form of StAR is partially unfolded, with the N-terminal domain entering the mitochondria and the partially unfolded C-terminus interacting with the OMM. Direct evidence has been presented showing that StAR exists as a molten globule. While certain native structure is retained at the N-terminal domain, the C-terminal domain folding appears to be less tight at the low pH that StAR may undergo on the mitochondrial membrane. Then, the tightly folded N-terminal domain could make StAR halt as it enters the mitochondria, extending the time window for the C-terminus to act.

Steroidogenic acute regulatory protein exhibits constitutive activity on the OMM, but no activity when localized to the intermembrane space (IMS) or to the matrix (107). Mitochondrial StAR protein import experiments using a modified leader peptide confirmed StAR exclusive activity on the OMM, as reflected by a negative correlation between the time of StAR mitochondrial entry and its activity. Once again, StAR role in promoting steroidogenesis is proportional to the time it spends on the OMM (107, 108).

Even when N-62 StAR form (which lacks mitochondrial peptide leader) does not access to the mitochondria, a few molecules of this protein are associated with the OMM as it is shown by immuno-electron microscopy (108, 109). This truncated StAR form would transport several cholesterol molecules, while complete StAR protein is able to bind just one. This suggests that StAR could transport several cholesterol molecules before entering mitochondria and be processed.

### Mitochondrial Dynamics and Its Regulation by Protein Kinases in Steroidogenic Cells

“Mitochondrial dynamics,” which includes fusion/fission events, is relevant for maintaining mitochondrial integrity. Indeed, mitochondrial plasticity is important for several cellular functions and for protection against aging-related changes. Among these functions, mitochondrial dynamics play a role in mitochondrial replication and repair, propagation of intramitochondrial calcium waves, and in the elimination, *via* mitophagy, of depolarized mitochondria (110). Two GTPases located on the OMM have a crucial role in mitochondrial fusion, mitofusin (Mfn) 1 and 2. These proteins, structurally related to dynamin, are expressed in several tissues, as brain (mainly Mfn2), liver, adrenal glands, and testis. Mfn1 and Mfn2 modulate the interactions mitochondria–mitochondria and endoplasmic reticulum (ER)–mitochondria and also mediate mitochondrial fusion acting in a concerted fashion with another GTPase located in the IMM, optic atrophy 1 (OPA1).

Mitochondria have been shown to be in constant movement within the cells, and this movement can be induced after steroidogenic hormone action. This event would allow the contact between mitochondria and other membranes. It is well known that the contact between mitochondria and ER plays an important role in cell metabolism and signaling transduction pathways. Indeed, it is considered as a unique subdomain termed the mitochondria-associated ER membrane (MAM), with a vast importance in regulation of Ca^2+^ signaling, mitochondrial bioenergetics, apoptosis, and lipid metabolism (111–113).

Mfn2 in the ER bridges mitochondria and ER by forming homotypic and heterotypic complexes, with Mfn2 or Mfn1 on the mitochondrial surface. Therefore, Mfn2 is critical for MAM formation by tethering ER to the mitochondria. A mitochondrial ubiquitin ligase, MITOL, has been described as the regulator of the ER–mitochondria interaction by controlling Mfn2 activity (114). Interestingly, Acsl4, the key enzyme involved in the regulation of steroidogenesis through AA release and induced by steroidogenic hormones (66, 79, 80), is localized and active in the MAM subdomain (115).

Dynamin-related protein 1 (Drp1) is required for mitochondrial fission. It is a cytosolic protein, which is recruited to the OMM by a poorly characterized multiprotein complex. In neurons, Drp1 phosphorylation by PKA in the mitochondria results in its inactivation and concomitant mitochondrial elongation (116). On the other hand, Drp1 phosphorylation by PKCδ at Ser579 increases mitochondrial fragmentation (117). In summary, several works support a key regulatory role for phosphorylation in mitochondrial morphology maintenance.

Although it is well recognized the relevance of mitochondrial dynamics in several cellular processes, its role in steroid synthesis is poorly described. Nevertheless, a work published 30 years ago described hormone-induced changes in intracellular location of the mitochondria and in the morphology of this organelle (118). Later, it was described that mitochondria move across the cell in a PKA-dependent manner after ACTH stimulation in H295R adrenocortical cells (119). This work demonstrates that ACTH/cAMP-stimulated mitochondrial movements depend on microtubules and have a role in the regulation of cortisol production, facilitating the shuttle of steroidoigenic substrates between the ER and mitochondria (119). In this cell line, the reduction of OPA1 facilitates the transfer of cytosolic Ca^2+^ signal into the mitochondrial matrix (120), which results in turn in enhanced aldosterone production (121). The authors stated that this is probably due to the altered diffusion conditions under OPA1 knockout. The study of an extramitochondrial form of OPA1 closely related to the lipid droplets ruled out any role of this fraction of OPA1 in cAMP-mediated steroid hormone production, the specific biological function of adrenocortical cells (66, 120). Moreover, the reduction of OPA1 in Leydig cells did not affect steroid production (98), suggesting that OPA1 is not critical for hormone-induced steroidogenesis. Then, the contribution of OPA1 and cristae remodelation to steroid synthesis needs further investigation.

### Mitochondrial Dynamics and Steroidogenesis

Steroid synthesis requires mitochondrial fusion induced by in a hormone-dependent fashion (89). The fact that Mfn2 is rapidly induced after the steroidogenic stimuli, and that blocking mitochondrial fusion by Mfn 2 knockdown expression reduce steroid synthesis, further supports a role of mitochondrial dynamics on steroidogenesis (89). The hormone-induced mitochondrial fusion might also be crucial for the generation of the mitochondrial multiprotein complex that facilitates the access of cholesterol to the P450scc system, since the mitochondrial rearrangement after cell stimulation is necessary for the relocalization of ERK1/2 to mitochondria. Moreover, the abrogation of mitochondrial fusion prevents the association of Acsl4 with the mitochondria, showing clearly that MAM formation depends on mitochondrial fusion (89). As previously mentioned in this review, SHP2 modulates mitochondrial fusion, suggesting that protein tyrosine dephosphorylation could be involved in the mechanism of mitochondrial dynamics (89). According to a published work (119), mitochondrial fusion might represent a limiting step in the onset of processes that require transport of intermediate products, e.g., liposoluble steroid hormones between organelles, probably mediated by MAM. In agreement with the previous results from our group, recent work demonstrate that hormone-induced MAM formation participates in the optimum transfer of cholesterol from the ER into the IMM increasing steroidogenesis rates (122). Then, steroid hormones might reach the plasma membrane without moving across the hydrophilic cytoplasm. Our group has shown that mitochondrial fusion is an essential process in regulating StAR mRNA levels and driving StAR to the mitochondrial context, probably participating in StAR mRNA stabilization and/or tethering the protein to the OMM (106) (Figure 2).

### Role of StAR Phosphorylation and Mitochondrial Fusion in StAR Localization

*In silico* molecular modeling has demonstrated that cholesterol binding to StAR could elicit a conformational change in StAR C-terminal domain, which in turn might favor the exposure of StAR Ser 232 and the docking domain for ERK. Therefore, StAR could be a substrate for ERK binding and phosphorylation, only when this protein is bound to cholesterol (42). This model is sustained by the fact that cholesterol binding to StAR promotes a decrease in its helical structure (123). The OMM is the most probable environment for the interaction between StAR and ERK since this submitochondrial domain anchors both StAR and ERK, as demonstrated in the previous work (42, 53). In turn, the overexpression of the mutated form of StAR, S232A, in steroidogenic cells prevents StAR phosphorylation by active ERK, thus proving that the kinase indeed phosphorylates this residue.

Cholesterol acts as an allosteric modulator of its own binding to StAR (123) and is strong stabilizing of the partially unfolded state in the StAR molecule (124). However, when cholesterol has to reach the P450scc, its release from StAR hydrophobic pocket is obligatory. Since ERK phosphorylation of StAR requires cholesterol, it is conceivable to think that StAR phosphorylation at Ser232 occurs after cholesterol binding. Thus, a conformational change in StAR induced by a negative charge at the Ser232 might reduce StAR affinity for cholesterol, favoring its release. This might in turn facilitate cholesterol transport into mitochondria to achieve high rates of pregnenolone synthesis.

Steroidogenic acute regulatory protein molecular structure has been partially studied (123–126), and the Ser232 residue is predicted to be localized in one of the last β barrels of the StAR-related lipid-transfer (START) domain (126, 127). It is well known that protein stability and interaction with several components are modulated by phosphorylation. Phosphorylation of proteins promotes acidic loops formation in their structure, as it has been described (128). The pH-dependent transition to the molten globule structure in the mitochondrial context (OMM) could provoke a weakened association between StAR C-terminal α-helix and lipid molecules, thus releasing cholesterol from StAR hydrophobic pocket. Under acidic pH conditions, the cholesterol affinity for START domain is significantly decreased (127). Thus, the addition of a phosphate group to StAR by ERK could establish a local decrease in pH, directing a conformational change in StAR, to a form with a lower affinity for cholesterol.

Our group has shown that StAR S232A expression significantly diminishes the localization of StAR in the mitochondria induced by hCG or cAMP. ERK phosphorylation affects mitochondrial StAR levels posttranscriptionally, as the expression of transfected StAR S232A is independent of cellular endogenous regulation (106). The mitochondrial module includes MEK, ERK, and cholesterol with a direct physical association between StAR and ERK (42). Their interaction facilitates StAR phosphorylation by ERK. Therefore, it could lead to phospho-StAR retention in the mitochondria, particularly on the OMM where ERK resides (53) (Figure 2).

Steroidogenic acute regulatory protein activity is determined by its localization on the OMM, and not its cleavage from the 37- to 30-kDa form (107). Hence, the longest StAR retention time on the OMM might render the maximal StAR activity in cholesterol transport, in agreement with the previous data (102). As described above, ERK is transiently activated after hormone stimulation in MA-10 cells (42). Its dephosphorylation could be mediated by MKP-1, since the temporal profile of mitochondrial MKP-1 and ERK dephosphorylation are compatible (95). Mitochondrial phospho-StAR and ERK interaction could avoid ERK dephosphorylation and inactivation. The temporal frame of ERK activity in this organelle correlates with highest StAR activity and cholesterol transport after hormone stimulation. These results agree with the fact that MKP-1 downregulation leads an increase in progesterone levels (95). The hormone-dependent induction of Mfn2 and mitochondrial fusion play an essential role in localization of ERK and StAR on the OMM and on the steroidogenesis (89, 129).

Taken together, these results offer new insights into StAR regulation by kinases and phosphatases and their impact on StAR site of action. The phosphorylation–dephosphorylation of StAR would contribute to modulate its affinity for cholesterol and to increase pregnenolone synthesis with a few molecules of StAR. In this work, we have reviewed StAR mechanism of action on cholesterol transport to the P450scc to achieve maximal steroid production. We have also described the role of phosphorylation–dephosphorylation events and mitochondrial fusion as novel regulators of the localization of StAR protein in order to carry out its action in steroidogenic cells.

## Concluding Remarks

Serine/threonine phosphatases have an important role in the regulation of adrenocortical cell functions, mainly steroid synthesis. In this context, the participation of PKA and PKC appears relevant, as phosphorylation events mediated by these kinases are involved in the expression and activation of StAR. Although StAR activation mechanism has not been fully described, it is known to require hormonal action on mitochondrial dynamic. Studies from other and our laboratory show that MAPKs, particularly ERK1/2, play an important role in StAR induction as well as in its posttranslational regulation in the mitochondria. In addition, ACTH-activated ERK1/2 regulates adrenal cell proliferation.

A field scarcely described is the role of PTPs in steroidogenic cells. We presented data on PTP activation triggered by ACTH through a PKA-dependent mechanism. In this context, PTP SHP2 has a role in the stimulation of steroidogenesis involving Acsl4 protein induction. In turn, Acsl4 promotes AA release, StAR induction, and steroidogenesis. Moreover, SHP2 along with ERK could also have a role in steroidogenesis promoting mitochondrial fusion. MKPs, a group of dual activity phosphatases that inactivate MAPK, are also regulated by steroidogenic hormones at multiple levels. While MAPK activation is linked to steroid production activation and cell proliferation, MKP induction is associated with the turn-off of hormonal signal through MAPK inactivation and, consequently, the downregulation of ERK-dependent events.

## Author Contributions

Ernesto J. Podesta, Cristina Paz, Cecilia Poderoso and Ana F. Castillo wrote the paper. All authors provided critical revisions of the paper.

## Conflict of Interest Statement

The authors declare that the research was conducted in the absence of any commercial or financial relationships that could be construed as a potential conflict of interest.

## Footnotes

^1^http://expasy.org

^2^http://expasy.org/prosite/

## Abbreviations

AA, arachidonic acid; ACBD3, acyl CoA-binding domain 3; Acot 2, acyl-CoA thioesterase; Acsl4, acyl-CoA synthetase 4; ACTH, adrenocorticotropin; Ang II, angiotensin II; ERK1/2, extracellular signal-regulated kinases 1 and 2; LH, luteinizing hormone; MAM, mitochondria-associated ER membrane; MAPKs, mitogen-activated protein kinases; MEK1/2, mitogen-activated protein kinase kinase 1 and 2; Mfn, mitofusin; MAKP, mitogen-activated protein kinase phosphatase; PAO, phenylarsine oxide; PKA, cAMP-dependent protein kinase; PKC, protein kinase C; PTPs, protein tyrosine phosphatases; PV, pervanadate; SHP2, Src homology domain 2-containing PTP; StAR, steroidogenic acute regulatory protein; ZF, zona fasciculata.
